# Changes in the large carnivore community structure of the Judean Desert in connection to Holocene human settlement dynamics

**DOI:** 10.1038/s41598-021-82996-6

**Published:** 2021-02-11

**Authors:** Ignacio A. Lazagabaster, Micka Ullman, Roi Porat, Romi Halevi, Naomi Porat, Uri Davidovich, Nimrod Marom

**Affiliations:** 1 Museum für Naturkunde, Leibniz Institute for Research on Evolution and Biodiversity at the Humboldt University Berlin, Invalidenstrasse 43, 10115 Berlin, Germany; 2 Department of Maritime Civilizations, Charney School of Marine Science & Recanati Institute for Maritime Studies, University of Haifa, Haifa, Israel; 3 Institute of Archaeology, The Hebrew University of Jerusalem, Jerusalem, Israel; 4 Israel Geological Survey, Jerusalem, Israel

**Keywords:** Ecosystem ecology, Environmental impact, Palaeontology

## Abstract

Investigating historical anthropogenic impacts on faunal communities is key to understanding present patterns of biodiversity and holds important implications for conservation biology. While several studies have demonstrated the human role in the extinction of large herbivores, effective methods to study human interference on large carnivores in the past are limited by the small number of carnivoran remains in the paleozoological record. Here, we integrate a systematic paleozoological survey of biogenic cave assemblages with the archaeological and paleoenvironmental records of the Judean Desert, to reveal historical changes in the large carnivore community. Our results show a late Holocene (~ 3400 years ago) faunal reassembly characterized by the diminishment of the dominant large carnivoran, the Arabian leopard (*Panthera pardus* sbsp. *nimr*), and the spread of the Syrian striped hyena (*Hyaena hyaena* sbsp. *syriaca*). We suggest that increased hunting pressure in combination with regional aridification were responsible for the decrease in the number of leopards, while the introduction of domestic animals and settlement refuse brought new scavenging opportunities for hyenas. The recent extirpation of leopards from the region has been a final note to the Holocene human impact on the ecosystem.

## Introduction

Evidence of present-day human influence on ecosystems suggests that ecological thinking should be informed by the study of late Pleistocene/Holocene anthropogenic involvement in the formation of extant biotic communities^1–3^. This knowledge is even more critical in semi-wild areas with relatively low human population pressure and a high potential for ecosystem recovery. Disentangling this anthropogenic effect on past ecosystems remains, however, a complex problem^4^. Current research suggests the negative impact of non-industrial and even hunter-gatherer societies on continental-scale trends of biodiversity loss, vegetation change, and megafaunal extinction^5–10^. The pace and scale of Holocene faunal extinctions have increased in connection to local cultural changes, human population growth, political instability, and environmental deterioration (e.g., aridification)^11–13^.

Most studies of human impact on animal communities in antiquity focus on large herbivorous fauna, common in archaeological deposits. Fewer studies on human impact on higher trophic levels^9,14–17^ are due to the rarity of contingent regional records of large carnivore faunas^18^, especially in the Holocene/Anthropocene. As a result, discussion on the relatively recent history of large carnivore communities is either theoretical or limited to dates of extirpation using first and last appearance data (FAD and LADs)^11,12^, with less emphasis on species- and region-specific histories of change^19^. A better understanding of the interaction between large carnivores, humans, and the environment in the Holocene is important because large carnivores are both keystone taxa and frequently hunted by humans^15,20^. Keystone predation on competitive dominants can increase diversity^21^ and the elimination of an apex predator might result in cascading top-down trophic effects in an ecological community^22–24^. The anthropogenic impact on large carnivores is accentuated in ecosystems with low biodiversity, in which trophic webs have fewer nodes^25^.

**Figure 1:**
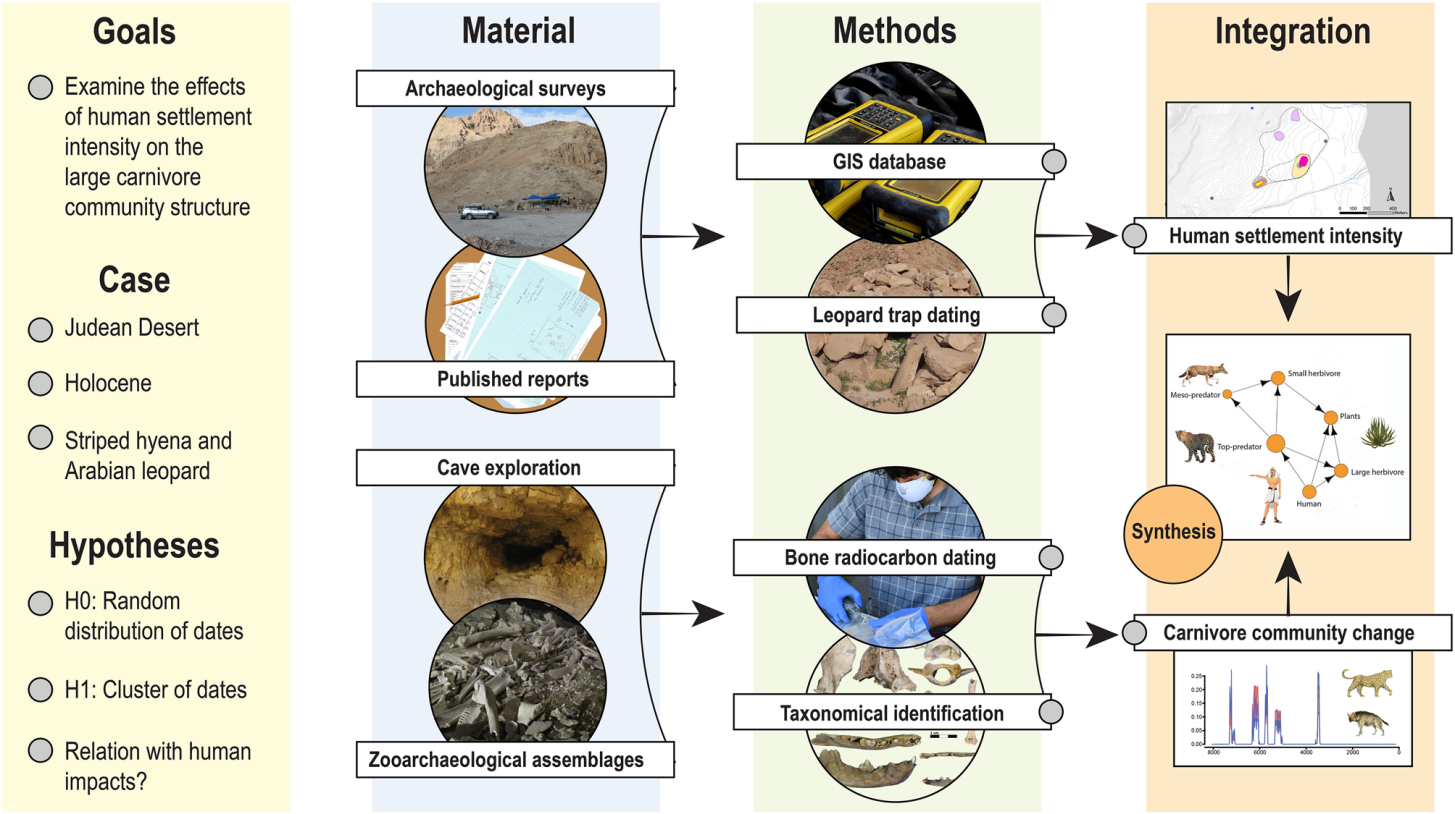
The research design followed in the study. Similar research strategies may be followed in other areas of the world with arid caves or where the zooarchaeological record is sparse.

**Figure 2:**
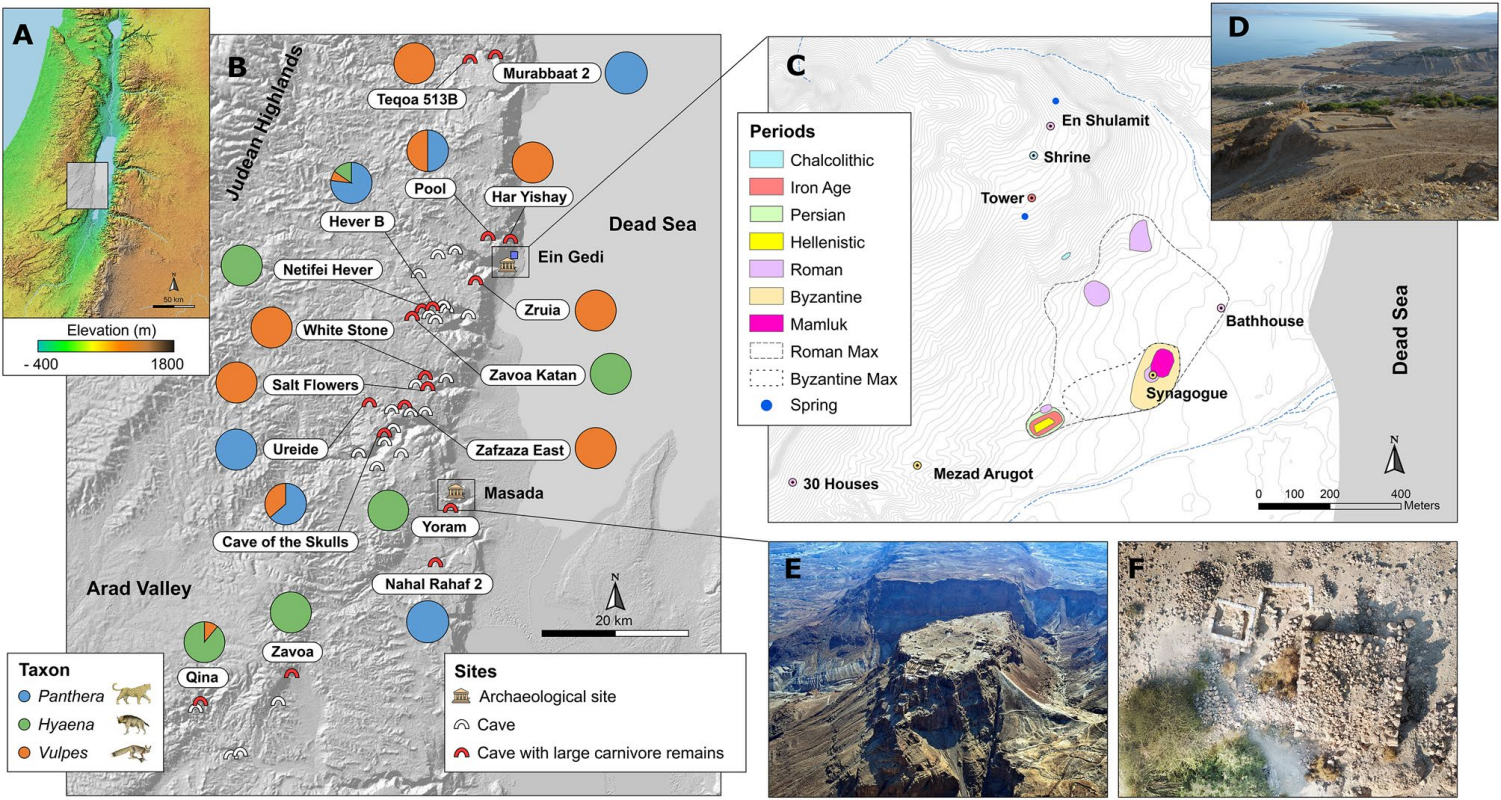
Map of the southern Judean Desert, showing caves and archaeological sites included in this study. (**A**) Elevation map of the Southern Levant. (**B**) Map of the study area showing caves surveyed (white arches) and caves where large carnivore remains were found and included in the statistical analyses (red arches). The pie charts show the relative proportion of leopard (*Panthera pardus*, blue), hyena (*Hyaena hyaena*, green), and fox (*Vulpes cana* and *V. rueppellii*, orange) remains recovered from each cave. (**C**) Topographic map of the Ein Gedi oasis, with convex hulls drawn around the archaeological sites of different historical time periods to calculate the area of occupation. (**D**) View of the Ein Gedi Oasis from the Chalcolithic shrine above the Ein Gedi spring. (**E**) Aerial view of the archaeological remains of Masada. Photo by Abraham Graicer used with permission. (**F**) Aerial view of the Roman to early Byzantine Tower in Ein Gedi during the 2019 field surveys, with several archaeological test pits around the main structure. Photo by Tal Rogovski used with permission.

**Figure 3:**
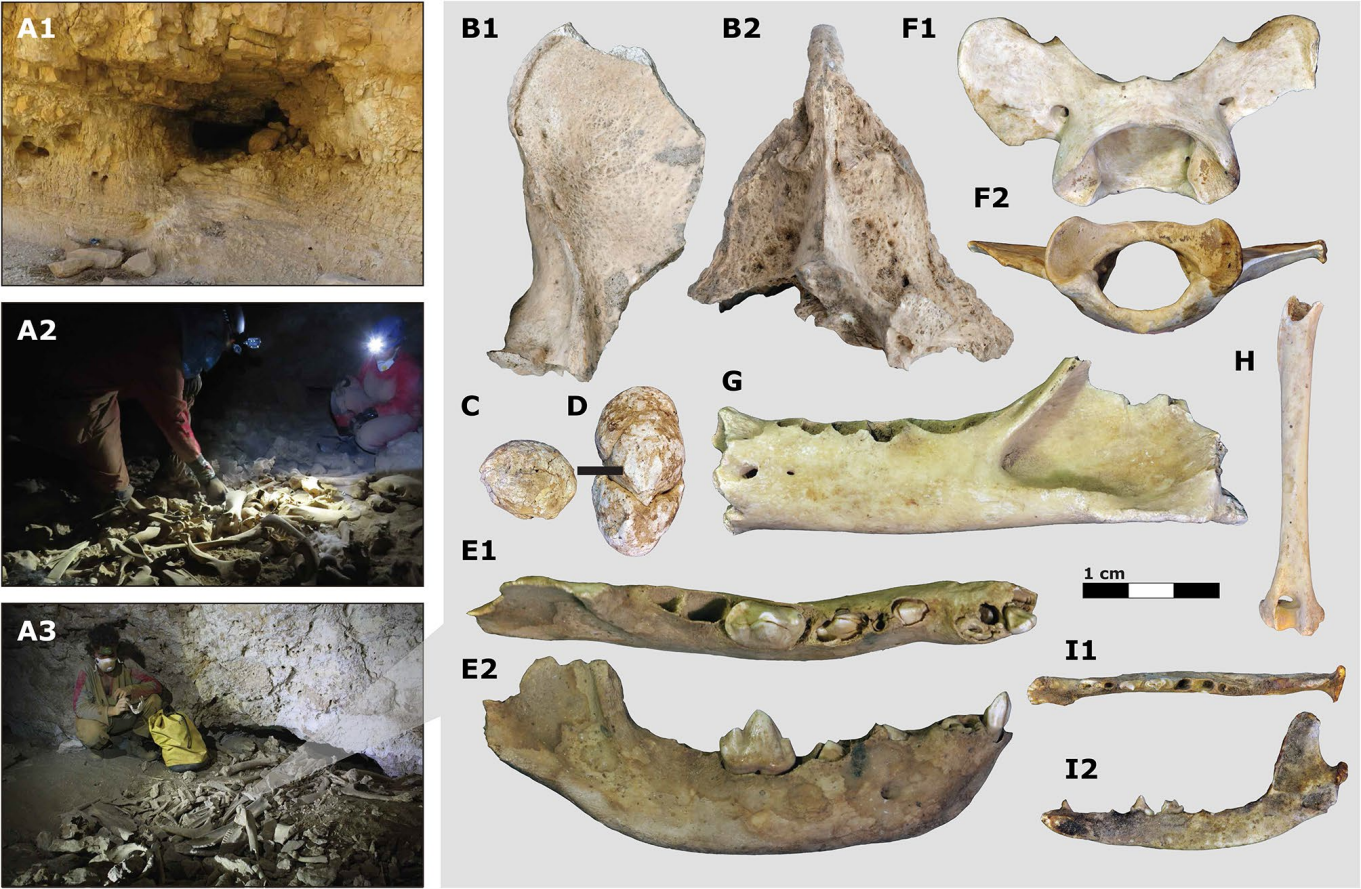
Large carnivore assemblages of the southern Judean Desert. Examples of carnivore dens and biogenic cave assemblages surveyed, including (**A1**) Zavoa Katan Cave, (**A2**) Zavoa Cave, and (**A3**) Qina Cave. Photo in A3 by Boaz Langford used with permission. Selected large carnivore specimens, including a *H. hyaena* occipital in (**B1**) lateral and (**B2**) posterior views, (**C**–**D**) *H. hyaena* coprolites, a juvenile right *H. hyaena* hemimandible in (**E1**) occlusal and (**E2**) labial views, a *P. pardus nimr* atlas in (**F1**) dorsal and (**F2**) inferior view, (**G**) *P. pardus nimr* left hemimandible in buccal view, (**H**) *V. cana* left humerus in posterior view, and *V. cana* left hemimandible in (**I1**) occlusal and (**I2**) buccal views.

Here, we apply a novel methodology to reconstruct changes in a regional large carnivore community in relation to human settlement dynamics throughout ~ 10 ky of the Holocene. To that end, we integrate data from multiple archaeological reports from the Ein Gedi region of the Judean Desert with a systematic paleozoological field survey of biogenic cave faunal accumulations in the area (Fig. 1). Radiocarbon dating of carnivoran remains is then used to explore temporal correlations with regional historical human activities and available paleoclimatic and paleoenvironmental records. The Judean Desert is a narrow strip of ca. 80 km long and 25 km wide which lies along the western border of the Dead Sea and the eastern flanks of the Judean Highlands. The southern border connects with the northeastern limit of the Negev Desert and its northern boundary coincides approximately with the northernmost point of the Dead Sea. The Judean Desert is particularly amenable to regional studies of faunal succession due to the many caves preserving a rich record of Holocene mammal bones (Figs. 2, 3). Furthermore, the Judean Desert in historical times had a relatively simple large (> 20 kg) carnivore community, comprising of three species: the Arabian leopard (*P. pardus nimr*), the Syrian striped hyena (*H. hyaena syriaca*), and the Arabian wolf (*Canis lupus arabs*)^26^, which makes potential change conspicuous and interpretable; and a well-defined history of human settlement, centered on the one major oasis in the area, at Ein Gedi^27^. Importantly, numerous paleoenvironmental proxies inform about the Mediterranean south Levantine Holocene climatic history, including pollen analyses, sedimentological records, δ^18^O curves and growth patterns from cave speleothems, and Dead Sea level fluctuations^28–32^. Therefore, changes in the carnivore community structure of the Judean Desert are framed within well-researched archaeological and climatic studies.

### Cave contexts

The caves of the Judean Desert have been little affected by fluvial and aeolian depositional processes throughout the later Pleistocene and Holocene^33,34^. The rarity of trees in the region makes caves and rock shelters an important nexus for the activity of leopards, hyenas, jackals, wolves, foxes, and porcupines, as well as smaller carnivores, rodents, and other non-mammalian fauna. Cave skeletal assemblages are composed of the faunal remains collected by different bone accumulators in the vicinity of caves and in situ mortality associated with cave-dwelling animals. Due to the continuous accumulation and alteration of biological material and the absence of significant sediment inflow, cave deposits are usually shallow (often < 1 m), consisting of bioturbated plant material and bones set in a matrix of—or covered by—decayed animal dung, sometimes mixed with anthropogenic deposits (Fig. 3). As a result, the cave skeletal assemblages as an aggregate preserve a time-averaged, non-stratified faunal record. Human food discards are often found but usually represent a small fraction of the assemblages. Some caves barely yielded any past human activity (e.g., Zavoa Katan, Aharoni, Salt Flowers, White Stone, Zavoa, Teqoa 513). The human exploitation of Judean Desert cliff caves during the Holocene was episodic. In those cases, the use of difficult-to-access caves in the region appears to have been as ephemeral hideouts for temporary refugees fleeing from the settled regions to the west at times of societal unrest and warfare (see Supplementary Information for references). Human activities in the caves certainly contributed to the mixing of anachronic remains. Unfortunately, widespread looting of caves in the last century have accentuated this pattern.

We assume that the shallow cave fills provide a time random, sparse sample of the large mammals that died naturally or that were hunted and scavenged throughout the deposit accumulation history. The sample is considered sparse because we expect that it represents only a small fraction of the natural death assemblage. We consider it time random for each cave due to the mixed temporal nature of the deposits^35^. We also make the explicit assumption that the taxonomic composition of the carnivore assemblage found in the caves (the death assemblage) reflects the proportion of these taxa in the region through time (the life assemblage), e.g., we expect to find more leopard remains during periods when leopards were more abundant and vice versa. Therefore, the distribution of dates obtained from carnivore remains reflects changes in their frequency through time, following a “dates as data” approach^36^.

## Research hypotheses

Clustering of carnivoran fauna dates from caves in specific segments of the Holocene timeline (H1) would suggest a change in the community structure that can then be discussed in relation to human settlement intensity and environmental change in the study region. We focus on leopards and hyenas because these taxa are known to interact with humans as predators and commensalists and are expected to respond to changes in human settlement intensity^37,38^. We also include fox remains (*Vulpes cana* and *V. rueppellii*), which are relatively abundant in caves, but we exclude wolves and jackals in our analyses because it is extremely difficult to separate their remains apart from those of local dog breeds. We especially expect hyena remains to be more numerous in periods when settlement intensity was highest because of the hyenas’ propensity to thrive on human settlement refuse^38,39^. Our null hypothesis (H0) is that the dates for each of the large carnivore taxa (leopard and hyena) will be distributed randomly throughout the Holocene if there has been no effect to the human settlement pulses and/or given a relatively stable climate.

## Results

### Human settlement intensity

Settlement intensity in the Ein Gedi oasis was estimated by calculating the area of the occupation of the archaeological sites in each historical time period. This information was gathered from published reports, with complementary surveys and small-scale excavations conducted by the authors to cover blank spots on the oasis survey maps. The area of occupation, combined with other archaeological findings, provides a relative quantitative measure of settlement intensity in the region throughout the Holocene.

**Figure 4:**
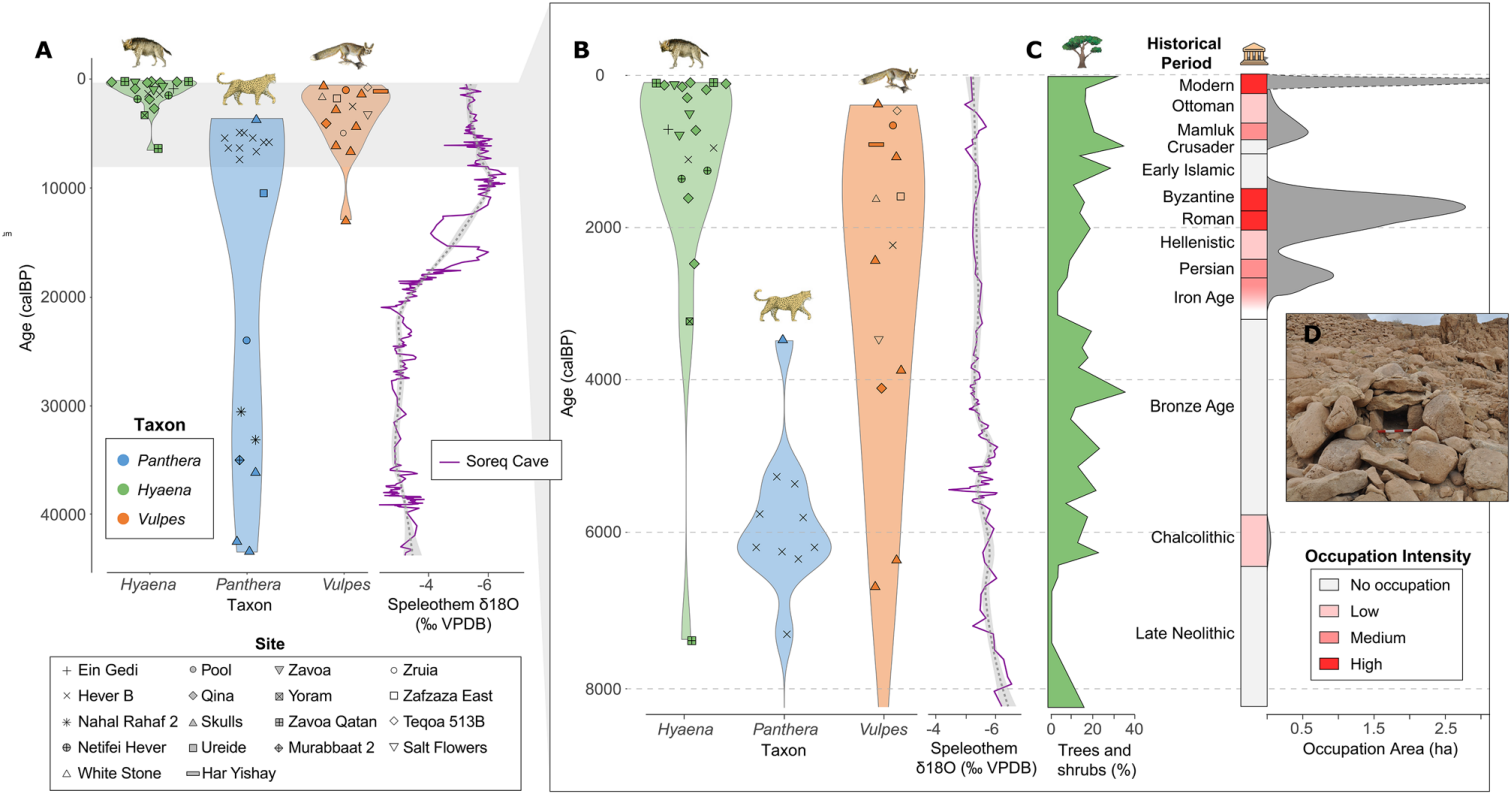
Distribution of radiocarbon dates by period, site, and taxon in relation to changes in local proxies for climate, vegetation, and human intensity in the Judean Desert. (**A**) Several leopard specimens were dated to the Late Pleistocene (~ 20,000–40,000 calBP) but (**B**) most of the remains are dated to the Holocene (< 4000 calBP). (**C**) Percentage of trees and shrubs derived from pollen data in ^29^ and human intensity calculated as settlement occupation area (Table 1). (**D**) Box trap (leopard trap) from Nahal Hever. Note that a large quantity of Dead Sea pollen may originate upstream the Jordan River, reflecting vegetation conditions further north. Occupation area calculated from drawing convex hulls around archaeological sites in the Ein Gedi oasis. Soreq δ^18^O data from^62^. The red star denotes the time when the Judean Desert was settled during the Iron Age, which is coincident with the disappearance of leopard remains and the increase of hyena remains in the zooarchaeological record.

Human settlement in the southern Judean Desert is limited to the few, isolated small oases along the west shore of the Dead Sea, of which only the oasis of Ein Gedi yielded the most substantial occupational remains (Fig. 2). The desert uplands were not settled for lack of water and food sources and were used intermittently by shepherds from the Judean Highlands to the west; by routes connecting settled regions on both sides of the Dead Sea Rift; and by nomads who did not leave substantial archaeological traces, until the Arab Bedouin settlement of the region from the eighteenth century CE onwards. Three distinct phases of human settlement can be observed at the oasis of Ein Gedi (see Supplementary Information for references). The first is dated to the late fifth millennium BCE (Chalcolithic period) and is best known for the isolated ritual complex above the major spring known as the Ein Gedi Shrine; several nodes of activity from the same period observed along the Ein Gedi spring slope suggest the existence of a sparsely populated hamlet at that period. The area extent of the archaeological sites dated to the Late Chalcolithic is small and we infer that human settlement intensity during this first phase of settlement was low (Figs. 2, 4; Table 1).

**Table 1:** Historical periods and human settlement intensity in the Ein Gedi Oasis, southern Judean Desert.

Period	Age range	Age BP lower	Age BP upper	Area (ha)	Max area (ha)	Occupation intensity
Late Neolithic	6400–4500BC	8350	6450			No occupation
Chalcolithic	4500–3800BC	6450	5750	0.04		Low
Bronze Age	3800–1150BC	5750	3100			No occupation
Iron Age I–IIB	1150–701BC	3100	2651			No occupation
Iron Age IIC	701–586BC	2651	2536	0.53		Medium
Persian	586–333BC	2536	2283	0.88		Medium
Hellenistic	333–63BC	2283	1887	0.17		Low
Roman	63BC–324CE	1887	1626	1.58	27.75	High
Byzantine	324–628CE	1626	1322	2.60	5.91	High
Early Islamic	628–1099CE	1322	851			No occupation
Crusader	1099–1291CE	851	659			No occupation
Mamluk	1291–1517CE	659	433	0.54		Medium
Ottoman (Bedouin)	1517–1917CE	433	33			Low

The area was calculated by drawing convex hulls around the main historical settlements.

The second phase of settlement in the Ein Gedi oasis occurred from the seventh century BCE (Iron Age) to the sixth century CE (Byzantine), with decadal-scale occupation gaps. During this span, Ein Gedi was a thriving community growing date palms and perfumes. Settlement remains are abundant, and agricultural terraces and water systems turned the oasis into an intensively managed landscape severely impacted by human activities, especially in the Roman-Byzantine era. Two small oases in the southwestern Dead Sea lakeshore, En Boqeq and En Tamar, feature small-scale agricultural estates and/or military outposts dated to the Roman and Byzantine periods. The area occupied by Roman settlements could have been as large as 27.7 hectares, and 5.9 hectares during the Byzantine period. Therefore, we infer that human settlement intensity during this second phase of occupation was high. After the Byzantine period settlement had come to an end, the oasis was resettled briefly during the fourteenth–fifteenth centuries CE (Mamluk period). Ein Gedi today is a kibbutz, and the entire oasis has been declared a nature reserve.

### Carnivore community structure

**Figure 5:**
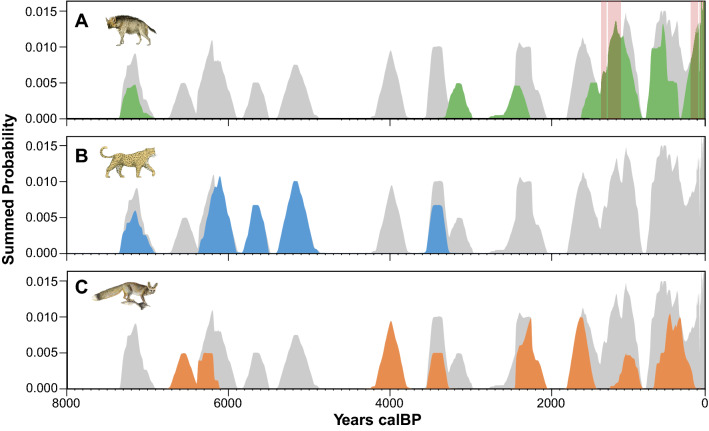
Summed probability density distribution of radiocarbon dates for each taxon. Significant deviations (*p* < 0.05) in the distribution of dates are highlighted with red bands, as is the case for hyenas (**A**). The dates are homogeneously distributed in the case of leopards (**B**) and foxes (**C**).

Out of 43 caves explored, 20 yielded remains of leopards, hyenas, and foxes, for a total of 65 specimens. These numbers reflect the inherent poor representation of these taxa in the zooarchaeological record. Our sampling has been, however, exhaustive; all identifiable large carnivore remains have been dated. These include 23 striped hyena (*H. hyaena*) bones and scats representing at least 22 independent occurrences (i.e., MNI); 23 leopards (*P. pardus*), representing at least 18 independent occurrences; and 19 foxes, 16 of which are Blandford’s fox (*V. cana*) and 3 are Rüppel’s fox (*V. rueppellii*). The generic relative abundance of remains collected and dated for each cave is shown in Fig. 2B. The dates of four leopard specimens and one specimen of *V. cana* were beyond radiocarbon calibration and are older than ~ 50,000 years. Conversely, three hyena specimens and three specimens of *V. cana* yielded dates that were too young for radiocarbon calibration and are considered modern. The presence of both recent and old specimens in the cave assemblages recalls the temporal mixture of the deposits. All dates beyond radiocarbon calibration were removed from the statistical models and are not included in Figs. 4 and 5.

The distribution of dates shows a date of the last appearance for leopards at ~ 3400 calBP, and a first occurrence date of ~ 7200 calBP for striped hyenas (Fig. 4A,B). All the fox calibrated samples fall between the final Pleistocene (~ 12,900 calBP) and late Holocene (~ 400 calBP), and the dates are homogeneously distributed (Figs. 4, 5). The summed probability distributions (SPD) calculated for all leopard and hyena specimens (NISP) show a similar pattern of FADs and LADs to that observed in the violin plots while highlighting, by the gray-filled interval between the blue and black lines, the range of uncertainty due to the possible inter-dependence of some of the leopard and hyena specimen occurrences at approximately the same date at the same site (Supplementary Fig. 1). A random permutation test of the shape of the SPDs shows that the distribution of hyena radiocarbon dates is non-random (number of bins = 15, *p* = 0.005), with significant deviations occurring between 1459 and 1311 calBP and after 273 calBP (Fig. 5).

Our data do not support significant temporal clusters in leopard and fox remains. However, the last leopard date coincides with the increase in hyena dates in the Ein Gedi region of the Judean Desert zooarchaeological record ~ 3400 calBP, after their first appearance a few millennia earlier (Fig. 4B). This faunal reassembly is broadly correlated with the establishment of permanent human settlements in the Ein Gedi oasis during the late Iron Age, but also with a change towards drier and warmer conditions at ~ 3200 calBP (Fig. 4B,C).

## Discussion

The dated specimens that we recovered from the paleozoological survey show a clear pattern: restructuring of the large carnivore community has taken place at approximately the time when the Ein Gedi oasis was first settled intensively, in the early 1st millennium BCE^40^. Our results confirm that leopards have been an integral part of the regional carnivore community since at least the late Pleistocene. Due to the low sample size, we cannot rule out statistically that the leopard clustering is random, but the absence of leopard remains younger than 3400 calBP shows a decline in the probability of finding a leopard in the paleozoological record towards the later part of the Holocene. On the other hand, there is no evidence for the presence of hyenas in the Late Pleistocene. All dates we obtained for hyenas are concentrated in the regional record from 7200 calBP to the present, with the great majority of dates (19/20, 95%) post 3400 calBP. The probability of finding a hyena post-dating the settling of the Judean Desert during the Iron Age is ten times higher than during the earlier parts of the Holocene. The inverse correlation between leopard and hyena dates frequency is clear, as the ranges of dates hardly overlap. Despite the small sample size, changes do not appear to have affected the smaller Blanford’s and Rüppell's foxes, which are reclusive and do not tend to interact with humans^41^.

Most likely, striped hyenas were already present in the Judean Desert periphery (including the Negev Desert to the south) but probably in lower numbers before human intensification began. Hyena remains have been reported from the Pleistocene site of Oumm Qatafa in the northern Judean Desert^42^ and from the Neolithic sites of Ashqelon and Qatif Y3 in the southern coastal plain^43^. Striped hyenas rely on large areas to support their resource requirements. In the Negev Desert, the average home-range size is ~ 60 km^2^ and the population density is 8.5 individuals/100 km^2^^44^. Nowadays, hyenas (both *H. hyaena* and *C. crocuta*) can survive in low-productivity habitats thanks to commensalism with humans, taking advantage of human trash, open landfills, and domestic animals^38,39^. We suggest that human settlements, and later nomad encampments, afforded ample foraging opportunities for these opportunistic scavengers, especially in the form of animal carcasses and butchery waste discarded at the periphery of settlements^39^.

Leopards, on the other hand, are not present in the cave record from the end of the second millennium BCE onwards, coinciding with the intensification of human settlement activities and with the first appearance of striped hyenas (Fig. 4B,C). The absence of leopard remains younger than 3400 calBP from the explored cave sites does not reflect the complete extirpation of leopards from the region. Rather, it shows a decline in the probability of finding a leopard towards the later part of the Holocene due to a decline in their population. Over a decade ago, genetic analyses of feces confirmed the presence of a male and two females in the Judean Desert, and four males and one female in the Negev Highlands^45^. However, the last individuals in the Judean Desert have since died, and now the leopards are considered extinct in the region. Leopards are persecuted when they prey on livestock and as a hunting trophy, but their recent decline is also caused by habitat loss and hunting pressure on prey species^37^. It may be suggested that hunting for prestige and protection of livestock has depressed leopard populations from the time a permanent human settlement system was founded in the region.

Leopard traps in the region are especially telling because they provide direct evidence to the involvement of humans in leopard hunting (Fig. 4D). Numerous leopard traps (n = 16) have been found in the vicinity of Ein Gedi^46,47^. Their location suggests that they were used to avoid leopard infiltration in the oasis^47^. The leopard traps consist of oblong piles of fieldstones (ca. 2.5–4 m. long; ca. 2 m. wide) and a roof made of large flat stones; these are known as box traps (Supplementary Figs. S1 and S2). There is an entrance for the leopard on one side and a smaller opening on the other side which is used to insert a weapon and kill the animal. Preliminary results from dating these traps (N = 5) in the study area suggest that most of them post-date the leopard LAD, supporting the case for more intensive hunting of leopards during the later Holocene. A desert kite was also found near Ein Gedi and was probably used to hunt gazelles. The combination of both hunting the leopard and its prey helps to explain the human impact on the leopard’s population in historical times^37^.

Climate change may have also played a role in the contraction of the Holocene leopard population in the Judean Desert, although the scale and magnitude of local Holocene climatic oscillations were smaller in comparison with those that occurred during the Pleistocene^28,48–51^. Regional paleoclimatic records obtained from the study of cave speleothems (e.g., Soreq Cave) indicate that the modern seasonal regime of wet winters and dry summers in the southern Levant has remained invariable since the beginning of the Holocene^52^. However, a drop in annual rainfall of about ∼ 200 mm on average occurred between 4600 and 4000 calBP^53^. These drier conditions were maintained for the rest of the Holocene, with fewer and relatively smaller oscillations ^53–55^. A relatively sharp drop in the Dead Sea level is documented at ∼ 3500 calBP^54,56–58^ and pollen records recovered from a sediment core drilled at the Ein Gedi shore suggest a change to drier and warmer conditions at ∼ 3200 calBP marked by the reduction of the tree and shrub pollen^29^. The absence of travertine deposition during at least 2000 years in Moringa Cave, situated near the Ein Gedi spring, confirms that precipitation during the Late Holocene was not enough to support speleothem deposition in caves^30,32,59^.

Paleoenvironmental reconstructions of the Judean Desert derived from all these proxies should be taken with caution. The oxygen isotope ratios in the speleothems reflect primarily the east Mediterranean source-water composition^60,61^ and Holocene scale changes in weather systems from the Mediterranean had a weak effect on precipitation in the Judean Desert due to local conditions of rain-shadow^33,62^. The Dead Sea basin sedimentation reflects the hydrological conditions in a large drainage area so that the Dead Sea level is mostly controlled by the water input of its main tributary, the Jordan River, and by precipitation regimes in the Mediterranean Zone^63,64^. Similarly, a large part of the pollen from the Dead Sea was likely transported by the Jordan River or wind-blown from the Judean Highlands, thus reflecting more the conditions upstream than those of the Judean Desert^60,61^. The deposition of speleothems in Moringa cave towards the end of the 1st millennium BC was likely associated with the overflow of the En Gedi Spring (see Fig. 2) and a high stand of the Dead Sea level linked with increased precipitation in the Judean Hills to the west as well as northern Israel^31,32,56,57^. It is likely that the study area has been hyper-arid during the entire Holocene and increased rainfall in the Mediterranean zone had little impact on the environment^64^. However, the reduction in water influx from the western hills may have influenced the local availability of water and vegetation. The extension of arid areas could have been larger during periods of Mediterranean drought, reducing the amount of vegetation at the desert fringe. These peripheral changes could have hindered the availability of prey (e.g., gazelles, ibex) for leopards and thus, their effective population in the Judean Desert. It has been hypothesized that the increase in water inflow during the Roman and Byzantine periods could have led to the flourishing of human populations in the desert^57^.

It is likely, therefore, that it was the combined effect of general aridification trends of the later Holocene and increased human pressure that contributed to the reduction of leopard populations in the area. The endpoint of this trajectory is, unfortunately, known from the late twentieth century local extirpation of the leopard in the Judean Desert. The Arabian leopard *P. pardus nimr* is estimated to have lost 98% of its post-1750 historic range^37^. Our data suggest that the geographical range reduction and population decline of Arabian leopards started earlier, at least locally in areas with higher human impact. Today, the Arabian leopard (*L. pardus nimr*) is critically endangered, with an estimated population of 80–290 individuals and decreasing^37^.

The configuration of modern landscapes is the result of complex interaction through time between multiple processes, including climate change and human-induced impacts^1,3,4,11,17,65^. The synchronic change in carnivore community composition, climate change, and human settlement dynamics in the Holocene Judean Desert suggests that humans, through hunting, waste production, and other forms of niche construction, modified keystone, high-level nodes in food webs in antiquity, probably causing further changes to their environment through trophic cascades^11,20,23^. Understanding how the ecological history of threatened ecosystems unfolded is essential for conservation biology and planning, but is fraught with methodological challenges, especially when large carnivores are concerned. The combined use of settlement, environmental, and paleozoological data can be used to validate and map the extent of ecosystem changes in the Holocene and beyond. Because arid caves are distributed across many of the more fragile and intermittently inhabited regions of the globe (e.g., the Caucasus, Arabia), it should be possible to replicate the present research design to other regions (Fig. 1), thereby overcoming the chronic shortage of top predators in Holocene archaeological assemblages. We have established a temporal sequence from a mix of anachronic assemblages, constructing a true fossil record that was previously untraceable. By doing so, the study of top-down anthropogenic effects in the Holocene is paved, and the door is open to the incorporation of other tools, such as stable isotope analysis and ancient DNA, to examine community composition and trophic effects through time.

## Materials and methods

### Archaeological data

Over the past two decades, UD and RP have been engaged in multiple avenues of exploration concerning the Holocene archaeological record of the Judean Desert, with specific emphasis on regional exploitation patterns and settlement history. These venues included systematic cave and site surveys, excavation and dating of corrals located on desert uplands, and reanalysis of previous excavations and sub-regional surveys to achieve a nuanced version of regional history (see Supplementary Table S1 for references).

During 2019–2020, surveys headed by RP and UD at the Ein Gedi oasis within the framework of this study were aiming to refine our understanding of settlement intensity in the 5th millennium BCE to 1st millennium CE, complementing previous work. Fieldwork in Ein Gedi included (i) collection of datable surface materials from polygons (collection units) located at the base of the Ein Gedi slope; (ii) 16 probes, each 1 × 1 m, excavated randomly across the slope, and (iii) small-scale excavations in four localities within the oasis. Dating of archaeological contexts was based on pottery typo-technology typical of respective periods of occupation. Our results were combined with the results of past surveys and excavations in the oasis using ArcGIS software to calculate potential habitation area as a proxy to settlement intensity.

To quantify human intensity in the Ein Gedi area, we drew convex hulls around the main historical settlements and calculated the area (ha) as a relative approximation to the occupation extent in each historical time period. The occupation extent, together with the data derived from the archaeological record, was used to classify local human intensity into low (< 0.5 ha), medium (0.5–1.0 ha), and high (> 1.0 ha) (Table 1).

### Paleontological data

MU and RP surveyed over 43 caves in the southern Judean Desert during 2019–2020, assisted occasionally by the other authors (Supplementary Fig. S3). The survey concentrated on caves in which previous archaeological surveys reported bone accumulations^66^. Efforts were limited to caves that are approachable without rappelling because we targeted sites that could have been used by terrestrial carnivores rather than by birds of prey. All potentially diagnostic bones of mammals the size of hyrax and larger were collected from topsoil, cracks, and sediment pockets; to that were added samples of hyena scats, which are conspicuous and easy to identify. The samples were supplemented by hyena, leopard, and fox remains from excavations carried out by A Ganor, E Klein, RP, MU, and UD on behalf of the Israel Antiquities Authority and the Hebrew University at the Cave of the Skulls in 2016^67^, and by NM and O Barzilai at the Nahal Rahaf 2 rock shelter on behalf of the Israel Antiquities Authority and the Recanati Institute for Maritime Studies, the University of Haifa, during 2019. Of the 43 surveyed caves, 20 yielded bones of the three genera understudy, *Panthera*, *Hyaena*, and *Vulpes* Figs. 2 and 3; Supplementary Tables S2 and S3). One exception is EG-039, a H*. hyaena* specimen that was not recovered from a cave assemblage; it was found during archaeological excavations by G Hadas in Ein Gedi. All specimens were identified to species by IAL and NM, using the comparative collections of the Laboratory of Archaeozoology at the University of Haifa, the Steinhardt Museum of Natural History, Tel Aviv University, the Laboratory of Palaeontology and Archaeozoology at the Hebrew University of Jerusalem, and the French National Museum of Natural History (Paris).

### Radiocarbon dating

We focused on the striped hyena and the recently extinct Arabian leopard, but also dated Blanford’s fox (*V. cana*) and Rüppell’s fox (*V. rueppellii*) specimens to represent smaller carnivoran taxa that are expected to have interacted less intensively with humans (as either predator, game, or commensal animal). Local populations of the other carnivores in the region, wolves (*C. lupus*), and golden jackals (*C. aureus*), cannot be morphologically distinguished from local dogs by their bones and were therefore not included in the study. All reported specimens were recorded in a database and photographed before being sampled for radiocarbon (~ 250 mg). Selected specimens were also surface scanned before sampling.

Radiocarbon dating was carried out at the Oxford Radiocarbon Accelerator Unit (ORAU) (N = 23) and at the Center for Applied Isotope Studies (CAIS), University of Georgia (N = 42) (Supplementary Table S2). When possible, organic carbon from collagen was used for radiocarbon dating. If bones did not preserve collagen, the bioapatite fraction of the bone was targeted^68^. It is usually preferable to use collagen for radiocarbon dating because the inorganic fraction of the bone is susceptible to external contamination and molecular exchange with soil carbonates. In desert caves, however, radiocarbon bioapatite dating does not yield significantly different results from collagen radiocarbon dating^69^. The depositional context of the survey assemblages suggests little external sediment inflow and dry depositional environment, conditions less susceptible to mineral exchange and contamination of the apatite fraction. To test whether combining bioapatite dating and collagen dating could result in potential biases, we dated two specimens using both collagen and bioapatite. The dates obtained were comparable in both instances (specimen HE-010: ORAU radiocarbon years BP on collagen 4477 ± 26; UGAMS on bioapatite 4420 ± 20. Specimen HE-231: ORAU radiocarbon years BP on collagen 5312 ± 25; UGAMS on bioapatite 5260 ± 20).

For extended methods on radiocarbon dating, see Supplementary Information.

### Data analysis

Radiocarbon dates were calibrated using the CalibInt13 curve^70^ and converted to summed probability distributions using the r package ‘rcarbon’^71^. The minimum number of individuals (MNI) for each taxon was calculated based on chronological distance and recovery location between dated specimens of the same species: namely, specimens dated to the same calibrated 2-sigma interval originating at the same site were counted as MNI = 1. Random permutations were applied to the summed probability distributions (SPD). Mainly, the dates are grouped in different bins and then randomly reshuffled (N = 1,000). The resulting distribution of probabilities are z-transformed and local significant departures are considered when the observed SPD is outside of a 95% envelope of simulated data. A global significance test is also computed by comparing the total data outside the simulation envelope. Data analyses and visualization were implemented in R (3.6.1) (R Core Team 2018) using package rcarbon^71^, and are accessible through GitHub (https://github.com/nmar79/leopards_hyenas.git). Maps were prepared using QGIS 3.12.

## Supplementary Information

Supplementary Information
