# Supplementary material for: Changes in the large carnivore community structure of the Judean Desert in connection to Holocene human settlement dynamics
Source: Sci Rep. 2021 Feb 11;11:3548. doi: 10.1038/s41598-021-82996-6 (PMC7878878; doi:10.1038/s41598-021-82996-6)
Supplement: Supplementary file 1 — Supplementary Information [file 41598_2021_82996_MOESM1_ESM.docx]

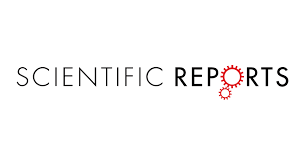


**Supplementary Material for**

Changes in the large carnivore community structure of the Judean Desert in connection to Holocene human settlement dynamics

Ignacio A. Lazagabaster^1,2^*, Micka Ullman^3^, Roi Porat^3^, Romi Halevi^3^, Naomi Porat^4^, Uri Davidovich^3^, Nimrod Marom^2^

^1^Museum für Naturkunde, Leibniz Institute for Research on Evolution and Biodiversity at the Humboldt University Berlin, Invalidenstrasse 43, Berlin, 10115, Germany.

^2^Department of Maritime Civilizations, Charney School of Marine Science & Recanati Institute for Maritime Studies, University of Haifa, Haifa, Israel.

^3^Institute of Archaeology, The Hebrew University of Jerusalem, Jerusalem, Israel.

^4^Israel Geological Survey, Jerusalem, Israel.

*Corresponding author: Ignacio A. Lazagabaster

**Email:** ignacio.lazagabaster@mfn.berlin

**This PDF file includes:**

Supplementary text

Figures S1 to S3

Tables S1 to S3

SI References

**Supplementary Information Text**

**Radiocarbon dating.** The radiocarbon techniques follow the standard procedures at the dating labs. Selected bones were cleaned with a sandblaster or ultrasonic bath and turned into bone powder, either using a hammer or a diamond drill bit. For collagen radiocarbon analyses, sample pre-treatment consisted in the demineralization of the bone matrix using 0.5 M of hydrochloric acid (HCL), followed by the addition of 0.1 M sodium hydroxide. A second wash with 0.5 M of HCl was accompanied by thorough rinsing with ultrapure water. Samples were then weighed into tin capsules and measured for carbon and nitrogen through combustion. The pre-treatment and radiocarbon dating procedures are described in detail in [[1]](https://paperpile.com/c/NM98vt/rIRNw). Samples are then graphitized according to [[2]](https://paperpile.com/c/NM98vt/gcqlx) and dated on the ORAU HVEE AMS system, as described by [[3]](https://paperpile.com/c/NM98vt/C9VxQ).

For bioapatite radiocarbon analyses, the bone powder was treated with diluted 1N acetic acid to remove surface absorbed and secondary carbonates. Carbon dioxide from the secondary carbonates was collected and purified for analysis. The chemically cleaned sample was then reacted under vacuum with H3PO4 to dissolve the bone mineral and release carbon dioxide from bioapatite. The resulting carbon dioxide was cryogenically purified from the other reaction products and catalytically converted to graphite using the method of Vogel et al. (1984). Graphite 14C/13C ratios were measured using the CAIS 0.5 MeV accelerator mass spectrometer. The sample ratios were compared to the ratio measured from the Oxalic Acid I (NBS SRM 4990). The sample 13C/12C ratios were measured separately using a stable isotope ratio mass spectrometer and expressed as δ13C with respect to PDB, with an error of less than 0.1‰.


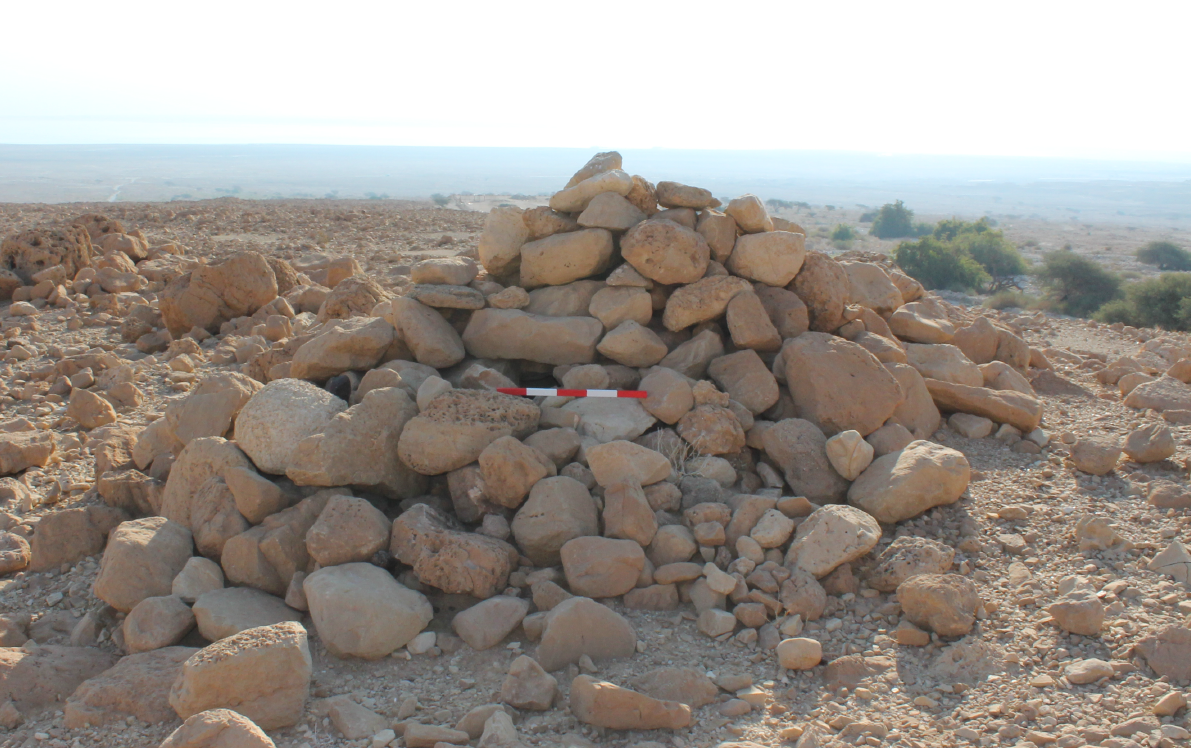


**Figure S1.** Box trap (leopard trap) in Nahal Mishmar.


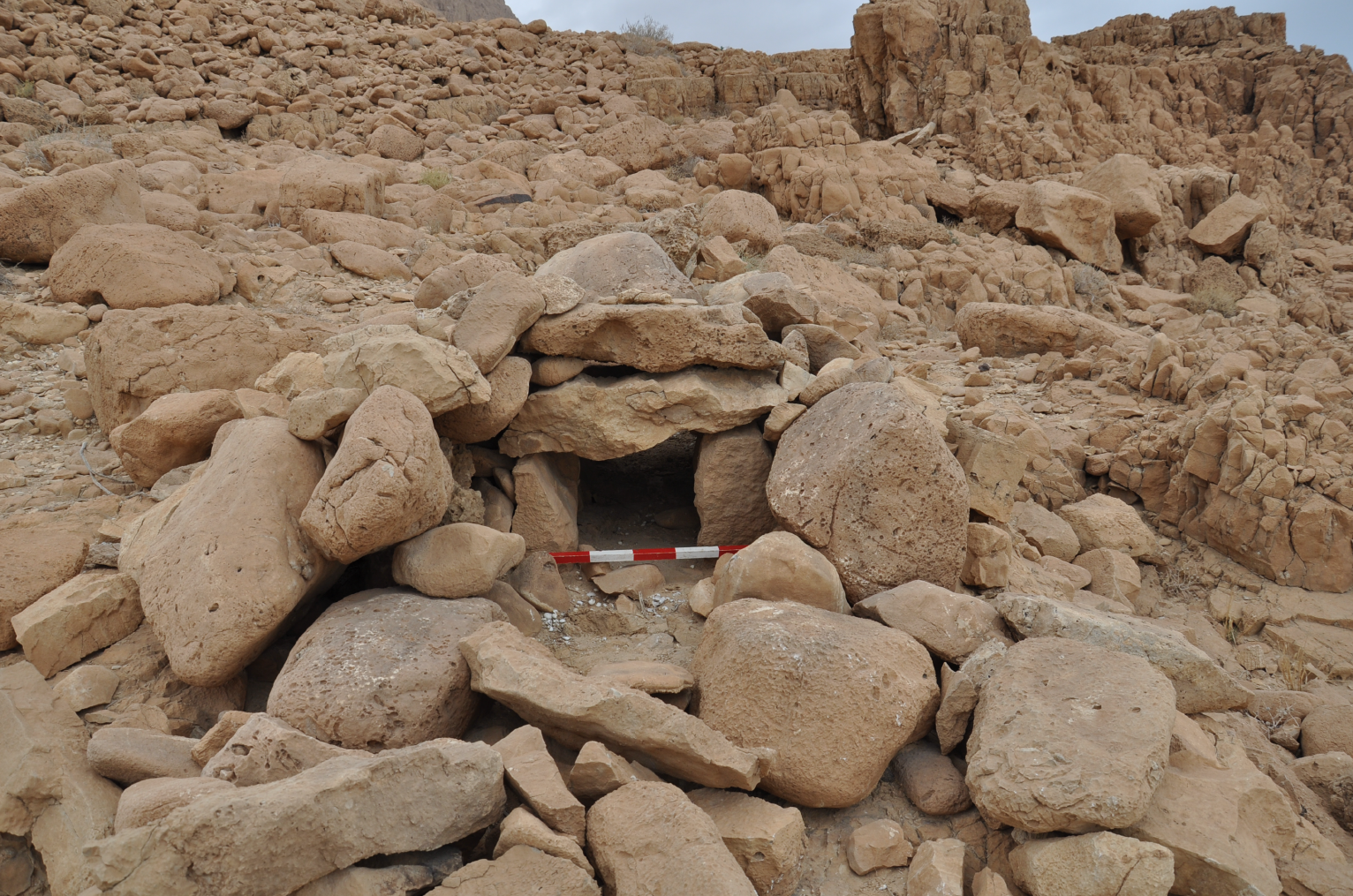


**Figure S2.** Box trap (leopard trap) in Nahal Hever.


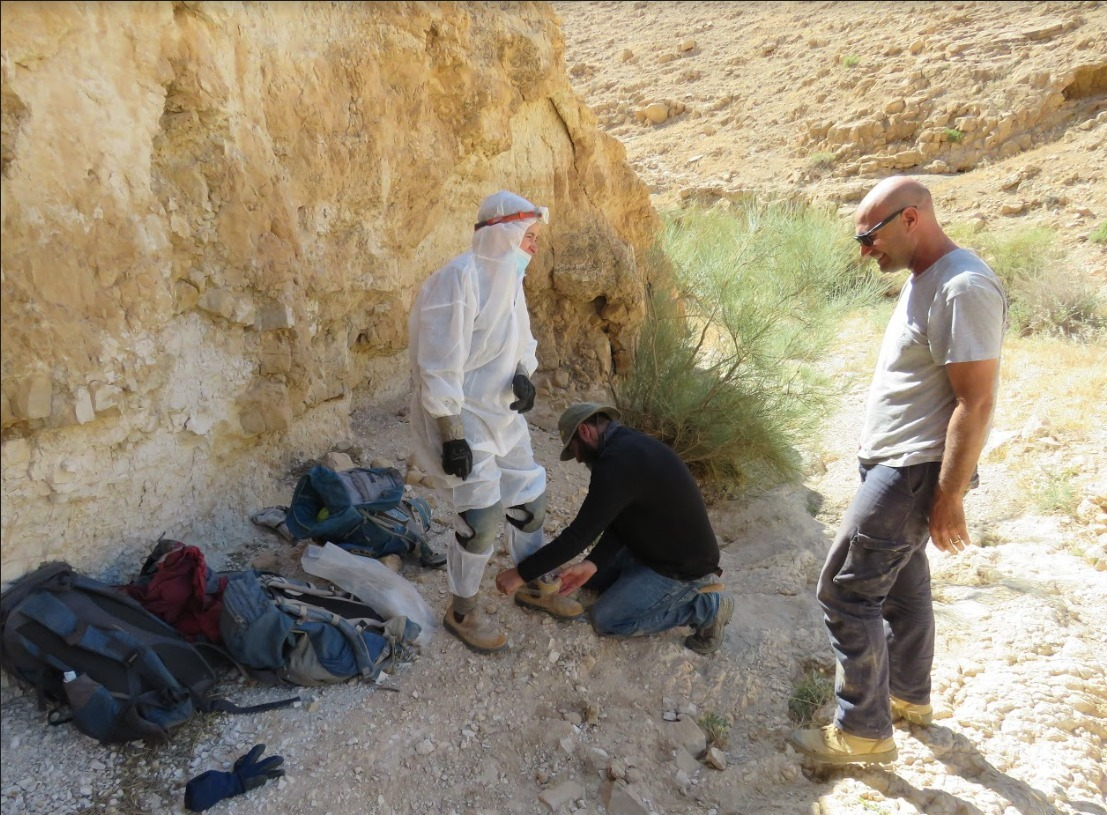


**Figure S3.** Author MU suiting up before entering Zavoa Katan Cave, helped by NM and UD, to protect herself against tick bites. Some caves in the region are still occupied by hyenas, porcupines, and medium-size mammals.

**Table S1.** Archaeological references of Ein Gedi.

| Period | Age Range | Age BP lower | Age BP upper | Occupation intensity | Refs. |
| --- | --- | --- | --- | --- | --- |
| Late Neolithic | 6400-4500BC | 8350 | 6450 | No occupation |  |
| Chalcolithic | 4500-3800BC | 6450 | 5750 | Low | [[4–9]](https://paperpile.com/c/NM98vt/P7d6s+Veubv+rqWSa+zTddw+O5kYp+2FYct) |
| Bronze Age | 3800-1150BC | 5750 | 3100 | No occupation | [[9]](https://paperpile.com/c/NM98vt/2FYct) |
| Iron Age I-IIB | 1150-701BC | 3100 | 2651 | No occupation | [[10,11]](https://paperpile.com/c/NM98vt/1F4Ql+HkHjv) |
| Iron Age IIC | 701-586BC | 2651 | 2536 | Medium | [[10,11]](https://paperpile.com/c/NM98vt/1F4Ql+HkHjv) |
| Persian | 586-333BC | 2536 | 2283 | Medium | [[8,10–12]](https://paperpile.com/c/NM98vt/O5kYp+HkHjv+wSR0k+1F4Ql) |
| Hellenistic | 333-63BC | 2283 | 1887 | Low | [[11]](https://paperpile.com/c/NM98vt/HkHjv) |
| Roman | 63BC-324CE | 1887 | 1626 | High | [[13,14]](https://paperpile.com/c/NM98vt/akLf0+gOF7G) |
| Byzantine | 324-628CE | 1626 | 1322 | High | [[12]](https://paperpile.com/c/NM98vt/wSR0k) |
| Early Islamic | 628-1099CE | 1322 | 851 | No occupation |  |
| Crusader | 1099-1291CE | 851 | 659 | No occupation |  |
| Mamluk | 1291-1517CE | 659 | 433 | Medium | [[12]](https://paperpile.com/c/NM98vt/wSR0k) |
| Ottoman (Bedouin) | 1517-1917CE | 433 | 33 | Low | [[15]](https://paperpile.com/c/NM98vt/7xhsr) |

**Table S2.** List of caves surveyed. Coordinates are in Israel Transverse Mercator.

| Caves | Latitude | Longitude |
| --- | --- | --- |
| Aharoni | 231590 | 593210 |
| Arugot 304 | 232000 | 597270 |
| Arugot 344 | 233020 | 597370 |
| Bar-Adon (506) | 235100 | 610700 |
| Dorban | 228030 | 584685 |
| DSES 117 | 237430 | 606350 |
| DSES 205 | 236740 | 606860 |
| DSES 75b | 235650 | 610730 |
| DSES 84 | 237340 | 606981 |
| DSES 86 | 236530 | 604950 |
| DSES 95 | 236810 | 606970 |
| Gev Zafzafa | 229810 | 586638 |
| Gorni | 229695 | 584020 |
| Happy Hyaenas | 229030 | 585346 |
| Har Yishai | 237060 | 597820 |
| Hemar | 218446 | 563091 |
| Hever B | 231605 | 593519 |
| Hever C | 232069 | 593436 |
| Hever East | 234140 | 593015 |
| Horror | 231700 | 593300 |
| Maale Naama | 227800 | 582650 |
| Murabaat 2 | 234660 | 610790 |
| Murabaat 4 | 235350 | 610700 |
| Nahal Rahaf 2 | 233380 | 577000 |
| Netifei Hever | 230840 | 593200 |
| Pool | 235500 | 597880 |
| Qina | 216065 | 566952 |
| Radum | 216058 | 566770 |
| Safrat as-Sana’ Channel | 230280 | 595970 |
| Salt Flowers | 230898 | 588265 |
| Shafan 45 | 226500 | 583740 |
| Skulls | 228930 | 585350 |
| Teenim C | 236435 | 605190 |
| Teqoa 513B | 235100 | 610700 |
| Ureide | 227900 | 586750 |
| White Stone | 231192 | 588360 |
| Yael | 229718 | 586736 |
| Yahel 8 | 221039 | 567180 |
| Yoram | 233615 | 580105 |
| Zafzafa East | 229960 | 586670 |
| Zavoa | 221996 | 568558 |
| Zavoa Katan | 230954 | 592746 |
| Zruia | 234670 | 595300 |

**Table S3.** List of specimens dated.

| Lab  name | Sample | Site name | ID | Material | Element | Species | Date | Error | CalBP | 2-sigma-low | 2-sigma-high |
| --- | --- | --- | --- | --- | --- | --- | --- | --- | --- | --- | --- |
| OxA | 39113 | Qina | QN-021 | collagen | vertebra | *H.hyena* | 96 | 19 | 117 | 256 | 225 |
| UGAMS | 48252 | Zavoa Qatan | TZQ-005 | collagen | mandible | *H.hyena* | 100 | 20 | 114 | 257 | 224 |
| OxA | 39177 | Zavoa | TS-006 | collagen | humerus | *H.hyena* | 120 | 19 | 105 | 265 | 218 |
| UGAMS | 46132 | Qina | QN-075 | TOC | scat | *H.hyena* | 130 | 20 | 107 | 268 | 211 |
| UGAMS | 48255 | Zavoa Qatan | TZQ-006 | TOC | scat | *H.hyena* | 140 | 20 | 113 | 278 | 239 |
| UGAMS | 46131 | Qina | QN-062 | TOC | scat | *H.hyena* | 150 | 20 | 145 | 281 | 249 |
| UGAMS | 46133 | Qina | QN-080 | TOC | scat | *H.hyena* | 190 | 20 | 183 | 290 | 264 |
| UGAMS | 46130 | Qina | QN-051 | TOC | scat | *H.hyena* | 200 | 20 | 181 | 294 | 266 |
| UGAMS | 46135 | Zavoa | TS-020 | TOC | scat | *H.hyena* | 500 | 20 | 525 | 542 | 509 |
| UGAMS | 46064 | Ein Gedi Hadas | EG-039 | bioapatite | cranium | *H.hyena* | 710 | 20 | 665 | 678 | 650 |
| OxA | 39253 | Qina | QN-023 | collagen | mandible | *H.hyena* | 724 | 19 | 670 | 682 | 654 |
| OxA | 39231 | Zavoa | TS-058 | collagen | mandible | *H.hyena* | 784 | 19 | 700 | 726 | 676 |
| UGAMS | 46124 | Hever B | HE-102 | TOC | scat | *H.hyena* | 1140 | 20 | 1021 | 1174 | 1167 |
| UGAMS | 46123 | Hever | HE-089 | TOC | scat | *H.hyena* | 1190 | 20 | 1109 | 1175 | 1158 |
| UGAMS | 48268 | Netifei Hever | NTH-002 | TOC | scat | *H.hyena* | 1280 | 20 | 1226 | 1277 | 1177 |
| UGAMS | 48269 | Netifei Hever | NTH-003 | TOC | scat | *H.hyena* | 1330 | 20 | 1273 | 1296 | 1262 |
| OxA | 39257 | Qina | QN-036 | collagen | mandible | *H.hyena* | 1614 | 20 | 1478 | 1535 | 1469 |
| OxA | 39258 | Qina | QN-048 | collagen | humerus | *H.hyena* | 2412 | 20 | 2422 | 2667 | 2658 |
| OxA | 39850 | Yoram | YO-003 | collagen | mandible | *H.hyena* | 2983 | 19 | 3163 | 3220 | 3074 |
| UGAMS | 48257 | Zavoa Qatan | TZQ-001 | bioapatite | mandible | *H.hyena* | 6260 | 30 | 7210 | 7261 | 7156 |
| UGAMS | 46122 | Christmas | CC-040 | TOC | Scat | *H.hyena* | Modern |  |  |  |  |
| UGAMS | 46125 | Hever B | HE-113 | TOC | Scat | *H.hyena* | Modern |  |  |  |  |
| UGAMS | 46118 | Tzeelim | TZE-001 | collagen | mandible | *H.hyena* | Modern |  |  |  |  |
| UGAMS | 44190 | Skulls | SK-703 | bioapatite | metatarsus | *P.pardus* | 3200 | 30 | 3417 | 3463 | 3463 |
| OxA | 38732 | Hever B | HE-010 | collagen | tibia | *P.pardus* | 4477 | 26 | 5174 | 5287 | 5156 |
| OxA | 47418 | Hever B | HE-053 | collagen | phalanx | *P.pardus* | 4508 | 30 | 5160 | 5303 | 5208 |
| OxA | 39109 | Hever B | HE-230 | collagen | mandible | *P.pardus* | 4935 | 26 | 5643 | 5718 | 5597 |
| OxA | 39107 | Hever B | HE-195 | collagen | pelvis | *P.pardus* | 4945 | 25 | 5652 | 5722 | 5599 |
| OxA | 39214 | Hever B | HE-008 | collagen | pelvis | *P.pardus* | 5301 | 25 | 6082 | 6187 | 6133 |
| OxA | 39110 | Hever B | HE-231 | collagen | tibia | *P.pardus* | 5312 | 25 | 6082 | 6188 | 6132 |
| OxA | 39091 | Hever B | HE-004 | collagen | radius | *P.pardus* | 5317 | 25 | 6083 | 6191 | 6098 |
| OxA | 47473 | Hever B | HE-232 | collagen | tibia | *P.pardus* | 5366 | 30 | 6174 | 6277 | 6232 |
| OxA | 47465 | Hever B | HE-124 | collagen | vertebra | *P.pardus* | 5375 | 28 | 6195 | 6279 | 6172 |
| OxA | 39216 | Hever B | HE-054 | collagen | metacarpus | *P.pardus* | 6239 | 26 | 7169 | 7253 | 7189 |
| UGAMS | 48266 | Ureide | UR-035 | bioapatite | femur | *P.pardus* | 8960 | 30 | 10150 | 10225 | 10117 |
| UGAMS | 44178 | Pool | EG-035 | bioapatite | metacarpus | *P.pardus* | 20040 | 40 | 24041 | 24212 | 23878 |
| UGAMS | 44183 | Nahal Rahaf 2 | NR2-006 | bioapatite | phalanx 1 | *P.pardus* | 26070 | 70 | 30247 | 30683 | 30623 |
| UGAMS | 44182 | Nahal Rahaf 2 | NR2-005 | bioapatite | phalanx 2 | *P.pardus* | 28680 | 70 | 33065 | 33376 | 32293 |
| UGAMS | 48282 | Murabaat 2 | MU-007 | collagen | phalanx | *P.pardus* | 30920 | 90 | 35296 | 35542 | 34870 |
| UGAMS | 44189 | Skulls | SK-702 | bioapatite | phalanx 2 | *P.pardus* | 31980 | 100 | 36298 | 36566 | 36079 |
| UGAMS | 44184 | Skulls | SK-164 | bioapatite | phalanx 1 | *P.pardus* | 39500 | 180 | 42834 | 43052 | 42619 |
| UGAMS | 44187 | Skulls | SK-605 | bioapatite | scaphoid | *P.pardus* | 40160 | 240 | 43266 | 43993 | 42880 |
| UGAMS | 44185 | Skulls | SK-200 | bioapatite | tibia | *P.pardus* | >49620 |  |  |  |  |
| UGAMS | 44186 | Skulls | SK-486 | bioapatite | phalanx 1 | *P.pardus* | >49620 |  |  |  |  |
| UGAMS | 44188 | Skulls | SK-701 | bioapatite | phalanx 1 | *P.pardus* | >49620 |  |  |  |  |
| UGAMS | 44181 | Murabbaat | MU-001 | bioapatite | ulna | *P.pardus* | >49620 |  |  |  |  |
| OxA | 39613 | Skulls | SK-239 | collagen | mandible | *V.cana* | 349 | 43 | 397 | 492 | 313 |
| UGAMS | 48263 | Teqoa 513B | C513-002 | collagen | mandible | *V.cana* | 390 | 20 | 471 | 503 | 438 |
| UGAMS | 46062 | Pool | EG-015 | collagen | mandible | *V.cana* | 580 | 20 | 605 | 639 | 590 |
| OxA | 39779 | Skulls | SK-047 | collagen | mandible | *V.cana* | 1143 | 19 | 1022 | 1174 | 1166 |
| UGAMS | 49005 | Har Yishai | HYS-004 | collagen | cranium | *V.cana* | 1150 | 20 | 1031 | 1174 | 1164 |
| UGAMS | 48258 | Zafzafa East | TZB-010 | collagen | mandible | *V.cana* | 1720 | 20 | 1603 | 1696 | 1662 |
| OxA | 39217 | Hever | HE-134 | collagen | mandible | *V.cana* | 2233 | 20 | 2222 | 2330 | 2296 |
| UGAMS | 46108 | Skulls | SK-877 | collagen | mandible | *V.cana* | 2320 | 20 | 2344 | 2356 | 2323 |
| OxA | 39611 | Skulls | SK-079 | collagen | mandible | *V.cana* | 3634 | 21 | 3944 | 4076 | 4042 |
| UGAMS | 46087 | Skulls | SK-220 | bioapatite | mandible | *V.cana* | 5480 | 20 | 6288 | 6306 | 6274 |
| UGAMS | 46106 | Skulls | SK-660 | collagen | mandible | *V.cana* | 5760 | 25 | 6559 | 6645 | 6489 |
| UGAMS | 44193 | Skulls | SK-094 | bioapatite | humerus | *V.cana* | 10970 | 30 | 12870 | 12989 | 12976 |
| UGAMS | 46103 | Skulls | SK-636 | bioapatite | mandible | *V.cana* | >49620 |  |  |  |  |
| UGAMS | 48264 | Aharoni | AHC-001 | collagen | skull | *V.cana* | Modern |  |  |  |  |
| UGAMS | 48265 | Zafzafa East | TZB-007 | collagen | skull | *V.cana* | Modern |  |  |  |  |
| UGAMS | 48259 | Zavoa Qatan | TZQ-004 | collagen | mandible | *V.cana* | Modern |  |  |  |  |
| UGAMS | 48274 | White Stone | CWS-005 | collagen | skull | *V.rueppellii* | 1770 | 20 | 1653 | 1713 | 1689 |
| OxA | 39578 | Salt Flowers | NM-013 | collagen | cranium | *V.rueppellii* | 3192 | 22 | 3414 | 3451 | 3373 |
| OxA | 39114 | Qina | QN-057 | collagen | mandible | *V.rueppellii* | 3694 | 23 | 4036 | 4142 | 4127 |

**SI References**

1. [Brock F, Higham T, Ditchfield P, Ramsey CB. 2010 Current pretreatment methods for ams radiocarbon dating at the oxford radiocarbon accelerator unit (orau). *Radiocarbon* (doi:](http://paperpile.com/b/NM98vt/rIRNw)[10.1017/S0033822200045069](http://dx.doi.org/10.1017/S0033822200045069)[)](http://paperpile.com/b/NM98vt/rIRNw)

2. [Dee M, Bronk Ramsey C. 2000 Refinement of graphite target production at ORAU. *Nucl. Instrum. Methods Phys. Res. B* **172**, 449–453. (doi:](http://paperpile.com/b/NM98vt/gcqlx)[10.1016/S0168-583X(00)00337-2](http://dx.doi.org/10.1016/S0168-583X(00)00337-2)[)](http://paperpile.com/b/NM98vt/gcqlx)

3. [Ramsey CB, Higham T, Leach P. 2004 Towards High-Precision AMS: Progress and Limitations. *Radiocarbon* **46**, 17–24. (doi:](http://paperpile.com/b/NM98vt/C9VxQ)[10.1017/S0033822200039308](http://dx.doi.org/10.1017/S0033822200039308)[)](http://paperpile.com/b/NM98vt/C9VxQ)

4. [Ussishkin D. 1980 The Ghassulian Shrine at En-gedi. *Tel Aviver Jahrb. Dtsch. Gesch.*](http://paperpile.com/b/NM98vt/P7d6s) **[7](http://paperpile.com/b/NM98vt/P7d6s)**[, 1–44. (doi:](http://paperpile.com/b/NM98vt/P7d6s)[10.1179/033443580788441071](http://dx.doi.org/10.1179/033443580788441071)[)](http://paperpile.com/b/NM98vt/P7d6s)

5. [Ussishkin D. 2014 The Chalcolithic Temple in Ein Gedi: Fifty Years after Its Discovery. *Near Eastern Archaeology* **77**, 15–26. (doi:](http://paperpile.com/b/NM98vt/Veubv)[10.5615/neareastarch.77.1.0015](http://dx.doi.org/10.5615/neareastarch.77.1.0015)[)](http://paperpile.com/b/NM98vt/Veubv)

6. [Shai Y, Porat R, Eshel H, Stern E. 2007 Moringa Cave. *En Gedi, Excavations I, Final Report (1961--1965), Israel Exploration Society, Jerusalem* , 391–403.](http://paperpile.com/b/NM98vt/rqWSa)

7. [Davidovich U. 2008 The Chalcolithic Period in the Judean Desert: Identification, Settlement Pattern and Material Culture as a Basis for Socio-economic Reconstruction. The Hebrew University, Jerusalem.](http://paperpile.com/b/NM98vt/zTddw)

8. [Davidovich U. 2014 The Judean Desert during the Chalcolithic, Bronze and Iron Ages (Sixth-First Millennia BC): Desert and Sown Relations in light of Activity Patterns in a Defined Desert Environment. The Hebrew University, Jerusalem.](http://paperpile.com/b/NM98vt/O5kYp)

9. [Davidovich U. 2013 The Chalcolithic - Early Bronze Age Transition: A View from the Judean Desert Caves, Southern Levant. *Paléorient* **39**, 125–138.](http://paperpile.com/b/NM98vt/2FYct)

10. [Māzār B, Dôtān Ṭrûdā, Dûnayevsqî İ. 1966 En-Gedi: the first and second seasons of excavations 1961-1962. *Atiqot/English series*](http://paperpile.com/b/NM98vt/1F4Ql)

11. [Stern E. 2007 *En Gedi Excavations I, Conducted by B. Mazar and I. Dunayevsky, Final Report (1961-1965)*. Israel Exploration Society, Institute of Archaeology, Hebrew University of Jerusalem.](http://paperpile.com/b/NM98vt/HkHjv)

12. [Hirschfeld Y. 2007 *En-Gedi excavations II: final report (1996-2002)*. Israel Exploration Society, Institute of Archaeology, Hebrew University of Jerusalem.](http://paperpile.com/b/NM98vt/wSR0k)

13. [Hadas G. 2012 Ancient agricultural irrigation systems in the oasis of Ein Gedi, Dead Sea, Israel. *J. Arid Environ.* **86**, 75–81. (doi:](http://paperpile.com/b/NM98vt/akLf0)[10.1016/j.jaridenv.2011.08.015](http://dx.doi.org/10.1016/j.jaridenv.2011.08.015)[)](http://paperpile.com/b/NM98vt/akLf0)

14. [Ben-Yehoshua S, Borowitz C, Ondrej Hanuš L. 2012 Frankincense, Myrrh, and Balm of Gilead: Ancient Spices of Southern Arabia and Judea. In *Horticultural Reviews* (ed J Janick), Wiley Online Library. (doi:](http://paperpile.com/b/NM98vt/gOF7G)[10.1002/9781118100592.ch1](http://dx.doi.org/10.1002/9781118100592.ch1)[)](http://paperpile.com/b/NM98vt/gOF7G)

15. [Frumkin A. 2015 Atlas of the Holey Land-Judean Desert Caves. *Magnes. Jerusalem, Hebrew*](http://paperpile.com/b/NM98vt/7xhsr)
